# Advances in Chemistry of Cosmetics

**DOI:** 10.3390/molecules31162850

**Published:** 2026-08-14

**Authors:** Chang-Gu Hyun

**Affiliations:** Department of Chemistry and Cosmetics, Jeju Inside Agency and Cosmetic Science Center, Jeju National University, Jeju 63243, Republic of Korea; cghyun@jejunu.ac.kr; Tel.: +82-64-754-1803

## 1. Introduction

The field of cosmetic chemistry has rapidly evolved from empirical ingredient discovery toward an interdisciplinary science integrating natural product chemistry, molecular biology, biotechnology, analytical chemistry, computational modeling, and translational dermatology. Consumers and regulatory agencies increasingly expect cosmetic ingredients to be supported not only by biological efficacy but also by mechanistic evidence, comprehensive safety evaluation, sustainability, and industrial feasibility. Consequently, contemporary cosmetic research emphasizes molecular targets, signaling pathways, systems biology, and clinically relevant validation rather than simple phenotypic observations. Against this background, the Special Issue ‘Advances in Chemistry of Cosmetics’ presents seven original studies that collectively illustrate how chemistry-driven research is reshaping next-generation cosmeceutical development [Contributions 1–7].

## 2. Coverage of Emerging Trends

One of the most striking themes emerging from this Special Issue is the convergence of sustainable natural resources, biotechnology, mechanistic pharmacology, computational chemistry, and safety science into a unified framework for cosmetic innovation. Rather than representing isolated investigations, the seven contributions collectively describe the evolution of cosmetic chemistry from empirical screening to evidence-based ingredient discovery.

Natural products continue to provide chemically diverse scaffolds for cosmetic applications. Studies on grape pomace and traditional medicinal plants demonstrate that agricultural by-products and ethnopharmacological resources remain invaluable sources of polyphenols such as flavonoids and anthocyanins, and other bioactive metabolites capable of promoting antioxidant protection, fibroblast regeneration, and skin repair [Contributions 1,4]. Importantly, these investigations also emphasize sustainability and circular economy concepts by transforming low-value biological resources into high-value cosmetic ingredients.

Beyond plant-derived materials, microbial biotechnology is emerging as an equally important source of functional cosmetic ingredients. Microbial exosomes derived from *Leuconostoc mesenteroides* simultaneously regulate inflammatory responses and melanogenesis through multiple signaling pathways, illustrating the growing importance of postbiotic technologies and extracellular vesicles as multifunctional cosmetic materials [Contribution 2]. Likewise, the anti-senescence activity of isoschaftoside demonstrates that understanding mitochondrial function, oxidative stress, and gene regulation is becoming central to anti-aging cosmetic research [Contribution 3].

Another dominant theme concerns the mechanism-based regulation of pigmentation. Drug repurposing using rifampicin [Contribution 5], together with mechanistic investigations of naturally occurring carboxylic acids and computational studies of hydroxyketone derivatives [Contributions 6,7], illustrates how depigmenting research is shifting from descriptive observations toward molecular intervention. These studies integrate enzyme kinetics, intracellular signaling, molecular docking, proteomics, toxicology, and cellular biology, thereby providing robust mechanistic evidence supporting future cosmetic applications.

**Figure 1:**
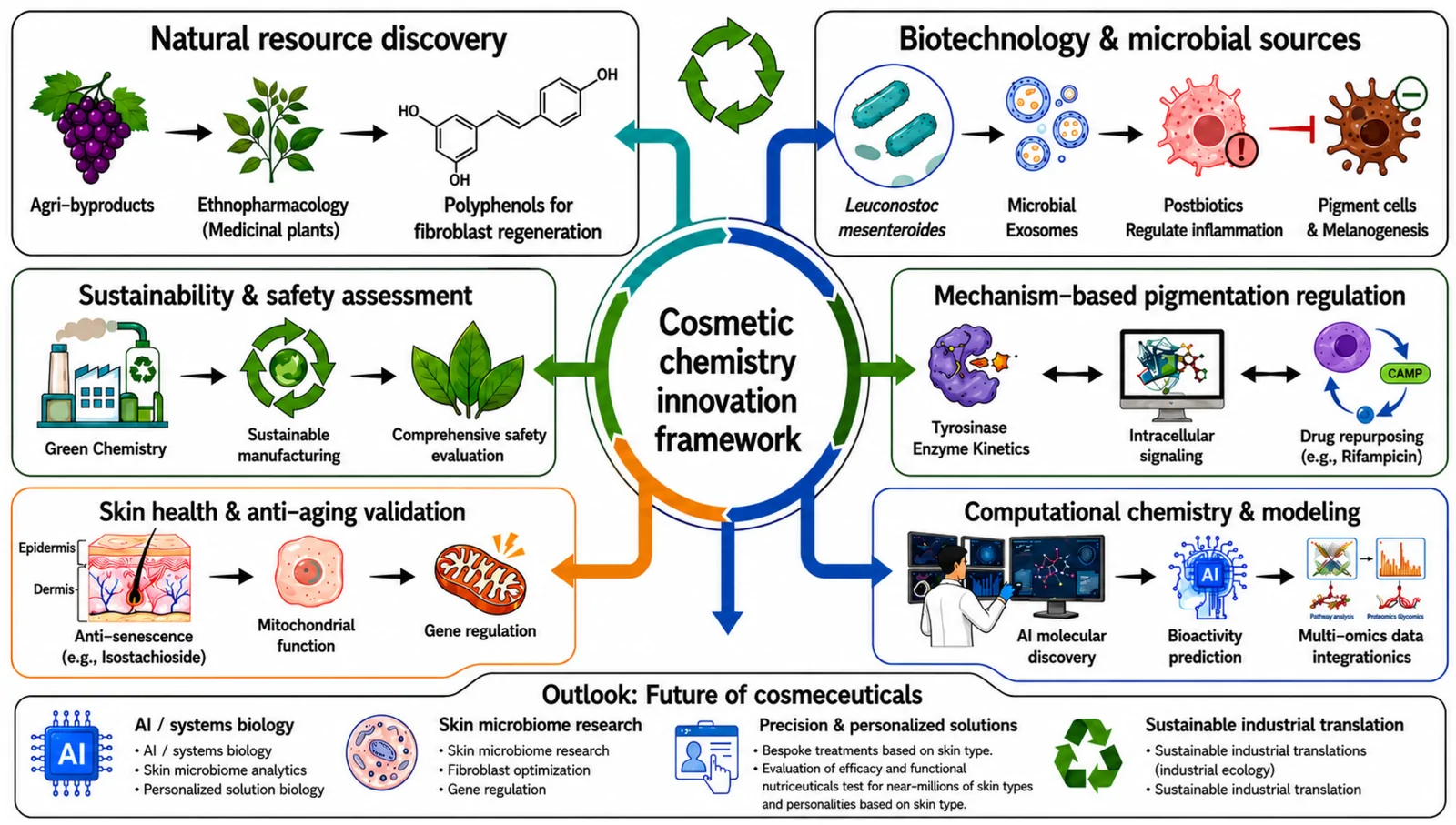
Integrated framework of chemistry-driven innovation in cosmetic and cosmeceutical research covered in this Special Issue. Schematic overview of the six research domains addressed in this Special Issue: natural resource discovery [Contributions 1,4], biotechnology and microbial sources [Contribution 2], mechanism-based pigmentation regulation [Contributions 5–7], computational chemistry and modeling, skin health and anti-aging validation [Contribution 3], and sustainability and safety assessment. The outlook panel summarizes future directions, including AI/systems biology, skin microbiome research, precision solutions, and sustainable industrial translation.

Collectively, these contributions highlight several emerging trends: sustainable ingredient discovery, integration of natural products and biotechnology, increasing reliance on computational chemistry, broader application of multi-omics technologies, and incorporation of rigorous safety assessment at early stages of ingredient development. Importantly, the studies complement one another by demonstrating successive stages of research in cosmetics, extending from resource discovery and bioactive molecule identification to mechanistic validation, efficacy assessment, safety evaluation, and translational application (Figure 1).

## 3. Conclusions and Outlook

The articles presented in this Special Issue collectively define the direction of future cosmetic chemistry rather than merely reporting individual advances. The field is increasingly characterized by multidisciplinary collaboration linking chemistry, biology, microbiology, pharmacology, materials science, computational modeling, and clinical dermatology. This integration enables the rational identification of bioactive molecules, clarification of molecular mechanisms, optimization of formulations, and acceleration of industrial translation.

Future progress will likely be driven by artificial intelligence-assisted molecular discovery, systems biology, multi-omics integration, precision formulation technologies, skin microbiome research, and advanced computational modeling. These approaches will facilitate prediction of biological activity, optimization of ingredient combinations, and identification of personalized therapeutic strategies. At the same time, green chemistry, sustainable manufacturing, and circular utilization of natural resources will become increasingly important in response to environmental challenges and consumer expectations.

The transition toward mechanism-based, evidence-driven cosmetic science represents a significant milestone for the discipline. By integrating sustainability, molecular mechanisms, biotechnology, computational chemistry, and clinical validation, next-generation cosmeceuticals will be better positioned to deliver safer, more effective, and scientifically substantiated products. Collectively, the contributions in this Special Issue provide an important foundation for this transition and offer valuable perspectives for future research in cosmetic chemistry and translational dermatological science.

